# Metabolic and Kinetic analyses of influenza production in perfusion HEK293 cell culture

**DOI:** 10.1186/1472-6750-11-84

**Published:** 2011-09-01

**Authors:** Emma Petiot, Danielle Jacob, Stephane Lanthier, Verena Lohr, Sven Ansorge, Amine A Kamen

**Affiliations:** 1 Biotechnology Research Institute. 6100 Royalmount Avenue, Montreal, H4P 2R2 Québec, Canada; 2 École Polytechnique de Montréal, Campus de l'Université de Montréal, 2500 chemin de Polytechnique, Montréal, H3T 1J4 Québec, Canada; 3 Max Planck Institute for Dynamics of Complex Technical Systems, Sandtorstrasse 1, 39106 Magdeburg, Germany

## Abstract

**Background:**

Cell culture-based production of influenza vaccine remains an attractive alternative to egg-based production. Short response time and high production yields are the key success factors for the broader adoption of cell culture technology for industrial manufacturing of pandemic and seasonal influenza vaccines. Recently, HEK293SF cells have been successfully used to produce influenza viruses, achieving hemagglutinin (HA) and infectious viral particle (IVP) titers in the highest ranges reported to date. In the same study, it was suggested that beyond 4 × 10^6 ^cells/mL, viral production was limited by a lack of nutrients or an accumulation of toxic products.

**Results:**

To further improve viral titers at high cell densities, perfusion culture mode was evaluated. Productivities of both perfusion and batch culture modes were compared at an infection cell density of 6 × 10^6 ^cells/mL. The metabolism, including glycolysis, glutaminolysis and amino acids utilization as well as physiological indicators such as viability and apoptosis were extensively documented for the two modes of culture before and after viral infection to identify potential metabolic limitations. A 3 L bioreactor with a perfusion rate of 0.5 vol/day allowed us to reach maximal titers of 3.3 × 10^11 ^IVP/mL and 4.0 logHA units/mL, corresponding to a total production of 1.0 × 10^15 ^IVP and 7.8 logHA units after 3 days post-infection. Overall, perfusion mode titers were higher by almost one order of magnitude over the batch culture mode of production. This improvement was associated with an activation of the cell metabolism as seen by a 1.5-fold and 4-fold higher consumption rates of glucose and glutamine respectively. A shift in the viral production kinetics was also observed leading to an accumulation of more viable cells with a higher specific production and causing an increase in the total volumetric production of infectious influenza particles.

**Conclusions:**

These results confirm that the HEK293SF cell is an excellent substrate for high yield production of influenza virus. Furthermore, there is great potential in further improving the production yields through better control of the cell culture environment and viral production kinetics. Once accomplished, this cell line can be promoted as an industrial platform for cost-effective manufacturing of the influenza seasonal vaccine as well as for periods of peak demand during pandemics.

## Background

In the last few years, the increasing risk of a pandemic influenza outbreak has brought into question the reactivity and efficiency of the present flu vaccine production mode. The current egg-based processes present different drawbacks, such as the minimum period of six months required after the selection of the flu strain to generate a sufficient supply of the vaccines. Consequently, pharmaceutical companies involved in influenza vaccine manufacturing are evaluating, among other expression systems, the cell-based mode of production as an attractive alternative to the hen's egg processes. Cell-based production processes are presently well-established technology platforms for manufacturing biopharmaceuticals, offer multiple advantages such as flexibility, expandability, and eventually shorter lead time.

Several cell-based processes using adherent cell lines, such as Vero or MDCK cells, for the production of influenza or other viral pathogens, are already well-documented [1-7]. However, adherent cell culture processes remain limited in cell density, due to microcarrier surface saturation. Also, they are mostly performed with serum-containing media [5]. Recently, new influenza production processes were proposed with suspension cell lines. Contrary to adherent cell cultures, suspension cell cultures have the potential to be operated at high cell densities and thus can thus achieve higher virus titers [5]. MDCK cells, which are the most popular cells used for influenza virus replication, were therefore adapted to suspension culture, resulting in a 1 logHA/mL increase of influenza titers over microcarrier MDCK cultures [4,8]. Other suspension growing cell lines, including duck AGE1.CR cells [9], human PER.C6 cells [10] or the avian embryonic derived stem cells EB14 (chicken) and EB66 (duck) [11], have been evaluated for infection and production of the different A and B influenza strains.

Previous work from our group demonstrated that the HEK293SF suspension cells are a valuable alternative for influenza production [12]. A scalable batch production process has been established producing 2.81 × 10^9 ^IVP/mL (IVP: infectious viral particles) and 4.01 log HA units/mL, values in the same range as the results obtained from MDCK or Vero cells [5,12]. Infection parameters, such as trypsin concentration and MOI (multiplicity of infection), were optimized to achieve high yields, but a leveling off of the maximal HA and infectious viral particles was observed at cell densities of infection higher than 4 × 10^6 ^cells/mL [12]. Although HEK293SF cells could grow in the selected serum-free medium to a maximal cell density of 10 × 10^6 ^cells/mL in batch mode [12], viral production appeared limited at a lower cell density. This so called "cell density effect" is consistent with observations previously reported by our group for other virus productions in HEK293SF cells. As for adenovirus production with this cell line, the limitations in viral productivity beyond a critical cell density could be related to either nutrient limitations or inhibiting by-product concentrations [13,14]. To alleviate these limitations as in the case of adenovirus production, different feeding strategies based on medium exchange [13], fed-batch mode [15,16] or perfusion strategies [17] have been explored.

Medium exchange is often used for viral productions despite the fact that this procedure is not easily scalable. For large-scale productions, the fed-batch strategy is the most convenient way to increase the cell density and alleviate nutrient limitations. However, extensive metabolic analyses are required to define an appropriate feed. In early stages of development, the perfusion strategy, although equipment and operation intensive, remains a valuable approach to alleviate metabolic limitations and reduce the residence time of the viral particles in the culture environment. Through continuous feeding of the medium, perfusion culture supplies cells with fresh nutrients and limits the accumulation of toxic by-products by dilution. Simultaneously, the viral particles produced are harvested in the perfused supernatant, thus avoiding a possible loss of functionality of the viral particles.

In the present study, a perfusion culture of HEK293SF cells was evaluated for high yield productions of a functional influenza virus. The primary objective of this work was to investigate if infection at high cell densities combined with a constant supply of fresh medium would increase the production yield. In addition, our secondary goal was to continuously harvest the influenza virus from the cell supernatant to minimize its residence time in the bioreactor and to maintain high infectious virus titer levels over the course of the production. Overall, the results led to a better understanding of the influenza virus production kinetics by HEK293SF cells under controlled culture conditions.

## Results & Discussion

The perfusion culture was compared to a batch culture in order to determine the differences if any in cell growth, and viability or in the metabolic pattern of HEK293SF cells due to continuous feeding of fresh medium. For both modes of operation the cultures were infected at a cell density of 6 × 10^6 ^cells/mL. This target cell density is generally in the late growth phase of HEK293SF cell batch culture. First, the study focused on analyzing the effects of the perfusion on the physiological state of the cell pre- and post-infection, and second, on establishing its impact on production yields once infected by an influenza H1N1 strain.

### Comparison of non infected batch and perfusion HEK293 cells cultures

#### Cell growth and death pattern

**Figure 1:**
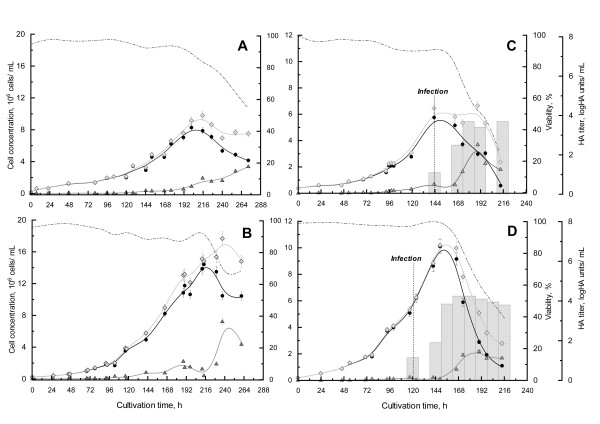
**Comparison of batch (A, C) and perfusion (B, D) culture processes for cell growth (A, B) and influenza production (C, D)**. Viable (*black circles*), dead (*up grey triangles*), and total (*grey diamonds*) cell densities were plotted with viability (*dashed lines*) and HA titers (*grey bars*) over time. The perfusion was started at 48 h of culture.

As expected, when perfusion was started after two days of culture at an exchange rate of 0.5 vol/day, the maximal cell density was significantly increased. Cell growth was maintained for 9 days, attaining a viable cell density of 15 × 10^6 ^cells/mL. In contrast, cell growth stopped in the batch system after 8 days of culture at a maximal cell density of 8.3 × 10^6 ^cells/mL (Figure 1A-B). Also, the specific growth rate, μ, was slightly higher in the perfusion system (0.024 h^-1 ^*vs *0.021 h^-1 ^for batch culture) (Table 1).

**Table 1:** Impact of feeding mode, batch or perfusion, and of influenza infection on HEK293SF cell growth and metabolism

	Standard deviation	Batch culture	Infected batch culture		Perfusion culture	Infected perfusion culture	
					
			*pre-infection*	*post-infection*		*pre-infection*	*post-infection*
**μ_max_**, h^-1^	10%	0.021	0.02	**-0.013**	0.024	0.023	**0.019**

**Y_Glc/X_**, mmol.10^-6^cell	17%	0.023	0.023	**0.026**	0.056	0.057	**0.038**

**Y_Lact/X_**, mmol.10^-6^cell	22%	0.012	0.014	**0.01**	0.088	0.083	**0.037**

**Y_Gln/X_**, mmol.10^-6^cell	10%	0.007	0.007	**0.001**	0.018	0.014	**0.01**

**Y_Amm/X_**, mmol.10^-6^cell	43%	0.001	0.001	**0.002**	0.003	0.003	**0.009**

**Y_Lact/Glc_**, mol.mol^-1^	19%	0.5	0.6	**0.4**	1.6	1.5	**1.0 **

**Y_Amm/Gln_**, mol.mol^-1^	43%	0.1	0.1	**2.0**	0.2	0.3	**1.1**

The values presented in bold letters correspond to culture phases with infection with A/PR/8/34 influenza strain.

**Figure 2:**
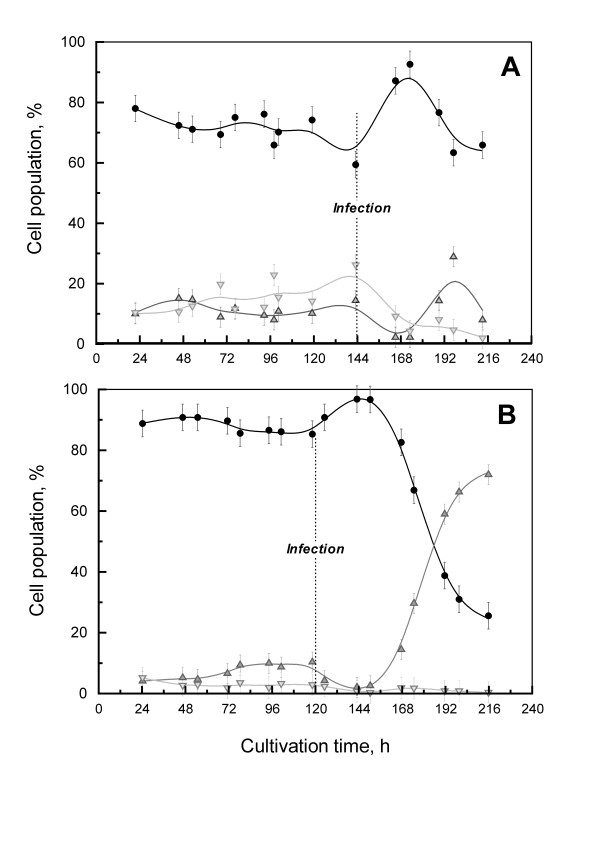
**Cell death pattern comparison for infected cultures (Batch A; Perfusion B)**. Viable (*black circles*), necrotic (*up grey triangles*) and apoptotic (*down grey triangles*) cell populations are presented. The perfusion was started at 48 h of culture.

A higher cell viability was observed for the perfusion culture at the targeted infection cell density (85% in perfusion *vs *60% in batch culture) (Figure 2). The apoptotic cell population was 23% lower in the perfusion culture than in the batch culture accounting for the higher cell viability in the perfusion culture. This observation is consistent with previously published results relating apoptosis to nutrient deprivation in cell cultures. For example, glucose or amino acid depletion have been related to the high rate of death in hybridoma cells. Also, depletion of growth factors or cytokines could also have a strong impact on cell death [18]. On the other hand, necrosis is generally described as occurring because of physical stresses or accumulation of high levels of toxic by-products, such as lactate or ammonium [19]. Consequently, the fact that perfusion provides fresh nutrients while clearing part of the toxic by-products in spent medium contribute in maintaining a high viable HEK293SF cell population in the culture.

#### HEK293SF cell metabolism

Metabolic and apoptotic pathways are strongly related as they converge on a shared set of proteins, as for example the GLUT transporter family or glycolytic hexokinase [20]. So, the metabolic states of the HEK293SF cells were evaluated and compared under batch and perfusion culture conditions in order to identify any potential changes in the physiological state of the cells pre-infection which in turn might impact the production capacity of the cells. Only the central metabolic pathways (glycolysis, glutaminolysis and amino acid pathways) were analyzed taking into account consumption and production of major carbon substrates and by-products. For consistency, the comparison of HEK293SF cell metabolism in perfusion versus batch culture was done between 50 h to 168 h, time periods corresponding to the exponential phase for both cultures once culture feeding was started for the perfusion culture. The global uptake and production per cells are presented in Table 1.

##### Glycolysis & Glutaminolysis

In the batch culture, the two major carbon substrates, glucose and glutamine, were not limiting nutrients since their concentrations at the end of the culture were of 7 mM and 0.54 mM respectively (corresponding to 21% and 13.5% of their initial concentrations). The maximum glucose and glutamine uptake rates in the batch culture were very close to the values previously reported in batch cultures of HEK293SF cells, with a ν_Glc max _of 0.13 mmol.h^-1^.10^-9^cell and a ν_Gln max _of 0.01 mmol.h^-1^.10^-9^cell [21-24]. In comparison with MDCK and Vero cells, the HEK293SF specific consumption rates were 5 to 7 times lower than those of MDCK cells (ν_Glc max _of 0.75 mmol.h^-1^.10^-9^cell) [25] and Vero cells (ν_Glc max _0.5 mmol.h^-1^.10^-9^cell) [26].

As previously reported, the two major metabolic by-products, lactate and ammonium ions, could be responsible for cell death [27]. However, under the culture conditions described herein, their concentrations never reached the toxic limits (20 mM for lactate and 3 mM for ammonia) described for other cell lines [27-30]. Their final concentrations were 9.9 mM and 1.7 mM, respectively, and their maximal production rates were of 0.15 mmol.h^-1^.10^-9 ^cell for π_Lact _and of 0.01 mmol.h^-1^.10^-9 ^cell for π_Amm_. These values are similar to the results obtained in other studies for HEK293 cells [21,23]. Interestingly, HEK293SF cells cultivated in HYQSFM TransFx medium appear to have a more efficient glycolytic metabolism in batch culture than MDCK or Vero cell lines, as the molar ratio Y_Lact/Glc _are 3 times lower than for these cell lines (0.5 vs 1.7 for Vero cells and vs 2.0 for MDCK cells [25,26]). These cell line metabolic characteristics will be further discussed in the section addressing the viral production aspect.

A direct impact of culture perfusion mode was observed on the glycolysis and glutaminolysis of HEK293SF cells. They consumed at least twice the amount of glucose and glutamine per cell as compared to the same phase in the batch culture (Table 1), leading to a depletion of glucose at the end of the perfusion culture. The differences between the two modes of culture are even more drastic regarding the specific by-product production rates. A 7-fold increase was observed for π_Lact _while the specific ammonia production, π_Amm _was enhanced of 3-fold (Table 1). These results are consistent with previous data obtained with the same cell line under similar operating conditions (0.5 vol/day perfusion rate) [17]. The molar ratios for Y_Lact/Glc _(1.6) and Y_Amm/Gln _(0.2) measured in this study also confirmed the ones previously obtained by Henry et al. [17] (1.6 and 0.4, respectively) and were also higher in comparison to the batch culture.

##### Amino acid metabolism

With the exception of aspartate, which was consumed at over 93% of its initial concentration in the batch culture, no clear amino acid limitations were observed for both the batch and perfusion cultures. In the perfusion culture, the final concentration of aspartate was 1.02 mM, a value 10 times higher than the final concentration in the batch culture. Consistent with previous reports on HEK293SF cell metabolism, aspartate and serine were the most-consumed amino acids. Aspartate as well as glutamate and serine, have already been described as limiting amino acids for HEK293SF cells cultivated in fed-batch mode [31]. Even so, we should note that, at the targeted cell density of infection (6 × 10^6 ^cells/mL), the aspartate concentration was 30% higher than its initial concentration. Thus, no amino acids were considered to be limiting at the time of infection or during the cell growth, either for the batch or the perfusion culture system. Glycine, alanine and cysteine, which could also be described as by-products of cell cultures, were the only amino acids to be released as reported for HEK293SF cells and other cell lines [26,32,33].

These comparative analyses support the conclusion that the HEK293 SF metabolic state is more active in the perfusion culture conditions. However, the quantification of key metabolites did not allow us to identify clear limitations or inhibitions that could account for the decrease in cell viability in the batch culture. It is likely that other molecules not quantified in the present study might be limiting the culture and inducing cell apoptosis in the batch culture [18].

#### Influenza production during batch and perfusion culture

In order to assess whether the perfusion mode of operation could overcome the leveling off of influenza production observed in our previous study for cell densities at infection higher than 4 × 10^6 ^cells/mL [12], cell cultures were infected at a target cell density of 6 × 10^6 ^cells/mL. Influenza production levels were thus monitored and the cell growth and death pattern as well as the metabolic state of the HEK293SF cells were compared in batch and perfusion cultures.

#### Cell growth and death pattern

Based on cell growth profiles only, the kinetics of the batch culture infected at 6 × 10^6 ^cells/mL were similar to the previous infection performed at 4 × 10^6 ^cells/mL by Le Ru et al., (2010) [12] (Figure 1-C). Cell growth arrest was observed after infection, and cell viability remained high for 24 hpi (hours post-infection). In contrast, the cell density in the perfusion culture after infection increased by 60% within 24 h, attaining 10 × 10^6 ^cells/mL (Figure 1 &2).

Influenza infection had an impact on the cell death pattern in both cultures. Cell apoptosis and necrosis were stopped for 24 hpi, then cell death increased dramatically during the next 48 h (Figure 2). This reduction of cell death right after infection with influenza viruses was also observed for AGE1.CR cells that were cultivated in Wave reactors [9]. A 24 h delay before an increase in cell death was also described for adherent MDCK cells infected with the A/PR/8/34 strain; however, the effect of the infection kinetics on cell viability appears to be highly dependent on the viral strain [34].

The mechanisms involved in apoptotic cell death due to influenza infection and the resulting production kinetics still remain only partially understood. Many studies concluded that influenza viruses induce apoptosis and provoke cell lysis, depending on the cell line studied. The time of occurrence of these events might vary between 10 to 40 hpi [35-37]. Also, NA and NS1 influenza proteins were reported to potentially regulate apoptosis during the viral replication cycle [35,37].

#### HEK293SF cell metabolism during influenza infection

To facilitate the comparison of HEK293 metabolic behavior, all of the cultures were divided into two phases: the growth phase without infection and the growth phase with infection. For consistency, the growth phase without infection started for batch and perfusion culture at 50 h, the starting time of the feeding in the perfusion system. It ends when the targeted cell density for infection, was attained (144 h for the batch culture and 120 h for the perfusion culture). Then, for both cultures, the growth phase with infection was reduced to 24 hpi, as this time period corresponds to the virus production phase before the inception of massive cell death (Figure 1C-D). This time period corresponds to the time between 144 and 168 h for the batch culture and between 120 and 144 h for the perfusion culture.

##### Glycolysis & Glutaminolysis

Consistent with observations made in non-infected cultures, the metabolic activity was higher in the perfusion than in the batch culture, both before and after influenza infection. For example, between the batch and perfusion growth phase with infection, Y_Glc/x _increased by 30% and Y_Lact/x _and Y_Amm/x _were 4 times higher (Table 1). Nevertheless, the increase of metabolic activity in perfusion compared to batch culture remains less pronounced in the infection phase than in the growth phase.

Furthermore, when comparing the cell metabolism before and after influenza infection, a decrease in lactate production accompanied by a constant or a slight decrease in specific glucose consumption was observed. Although this pattern has been already described in the case of MDCK cells infected with a H3N8 strain [38], this metabolic trend is not common. Other studies of the same group, performed with MDCK cells either in 5-L bioreactor [7] or in 6-well plate [39], has demonstrated a clear increase of the glycolytic pathway activity after 12 hpi. For these cases, the authors concluded that metabolic changes for MDCK cells undergoing influenza replication are to a lesser extent related to the virus replication itself, but rather specific to apoptosis inception occuring at the same time [39]. Interestingly, the metabolic behavior of MDCK cells seems to be dependent on the MOI employed. A reduction in glycolytic activity was observed for infection at low MOI [38] whereas high glycolytic behavior was observed for infections at high MOI [7,39]. In this context, it should be kept in mind that the HEK293 cell present a very different metabolism than other kidney cell lines used for influenza production such as MDCK or Vero cells. This is supported by differences in glucose consumption rates during normal growth without infection (see earlier section). It is thus very likely that the glycolytic response to influenza replication is cell line dependent.

In the case of HEK293SF cells, the molar ratio of Y_Lact/Glc _decreased by about 30% in both batch and perfused cultures after infection. Apparently, during the short growth phase from infection until 24 hpi, HEK293SF cells were using glucose more efficiently, with a larger part of this substrate being used either for growth or for viral production. By comparing these results with other viral productions in HEK293SF cells, it can be concluded that this metabolic behavior is specific to the virus produced: for example, in the case of adenovirus productions on HEK293SF cells in the perfusion system, the Y_Lact/Glc _ratio was increased by 12% [17].

With respect to the glutaminolysis pathway and the generation of ammonia, it is noticeable that for both infected cultures, the Y_Amm/Gln _ratio increased significantly after infection (batch 20-fold, perfusion 4-fold). This major increase for the batch culture is mainly due to an important decrease in the specific glutamine consumption (Y_Gln/x_), also corresponding to the growth phase plateau of the culture. It seems that the HEK293SF glutaminolysis metabolism is not favourable for virus multiplication as more ammonia was produced in both cultures. In fact, concentrations as low as 1 mM of ammonia can induce a reduction of 50% of the A/PR/8 strain production [39,40]. Indeed, ammonia is thought to be acting on virus intralysosomal pH, and therefore potentially affects the lysosome-dependent stage of the influenza infection process [40].

##### Amino acids

According to Sidorenko & Reichl [40,41], the intracellular pool of free amino acids could be a possible bottleneck for virus productions, as high virus yields require the uptake and synthesis of additional resources during the infection process. In our case, with a total initial amino acid concentration of 48 mM, the free amino acid content of our media is significantly higher than the usual amino acid content of serum-free media used for influenza productions (30.3 mM for Ex-Cell MDCK medium [4], 21.8 mM for SMIF8 medium and 16.2 mM for Episerf [9]). Therefore, it is not surprising that the amino acids quantification did not demonstrate any limitations at the time of infection for either the batch or the perfusion cultures. In addition, no limitations of amino acids were found during the 24 hpi growth phase.

In the batch culture, most of the free amino acid concentrations increased or presented a very low specific uptake over that period (Table 2). Only five amino acids presented an uptake higher than 5% of their initial concentrations (glutamine, histidine, proline, valine and methionine) with a maximal uptake for glutamine (15.3% of its initial medium content). In the perfusion culture, metabolic activity seemed to be increased compared to the batch culture, as most of the amino acids were consumed at higher rates (Table 2). Glutamine was also among the most highly consumed amino acid in the perfusion system (36% of its initial concentration) along with cysteine (48%), leucine (23%), lysine (21%) and arginine (22%). After 24 hpi, a massive release of amino acids is observed in both batch and perfusion cultures, which is certainly linked to cell lysis provoked by virus production.

**Table 2:** Free amino acid uptake and release during HEK293SF cell growth after infection.

		Gln	Asp	Glu	Ser	Asn	Gly	His	Thr	Arg	Ala	Pro	Cys	Val	Met	Ile	Leu	Lys	Phe
Free amino acid level before cell infection, mM	Batch	2.21	1.10	1.62	5.84	7.95	0.66	0.98	2.87	1.78	1.01	4.36	0.43	2.50	0.80	2.31	3.57	2.66	1.14
	
	Perfusion	1.77	0.87	1.48	2.80	7.06	0.73	0.87	2.17	1.32	1.28	3.70	0.02	2.04	0.67	1.91	3.11	2.17	0.96

Specific uptake or release of free amino acids, μmol.10^-6 ^cell	Batch	0.09	0.00	-0.03	0.01	-0.06	-0.03	0.01	-0.05	0.01	-0.09	0.05	-0.04	0.03	0.01	0.01	-0.01	-0.03	-0.01
	
	Perfusion	0.12	0.02	-0.01	0.08	0.00	-0.06	0.01	0.03	0.04	-0.08	0.00	0.02	0.03	0.01	0.05	0.09	0.06	0.01

Values for specific uptake and release were calculated between infection time and the start of cell death (24 h post-infection). Positive values represent amino acid uptake, while negative ones represent their release.

Overall, the differences observed between batch and perfusion amino acid uptake and release did not demonstrate a specific pattern for amino acid metabolism during influenza production. Secondly, if the free amino acid pool is in excess compared to the cell's requirements for virus synthesis, the only element impacting the amino acid uptake and release is the metabolic activity of the cell. This observation also leads to the hypothesis that any potential limitation and/or inhibition during the batch culture, leading to a leveling in viral production observed in previous studies with HEK293 cells [12], was due to other non-quantified components in the culture medium.

#### Production of Influenza virus A/PR/8/34

**Figure 3:**
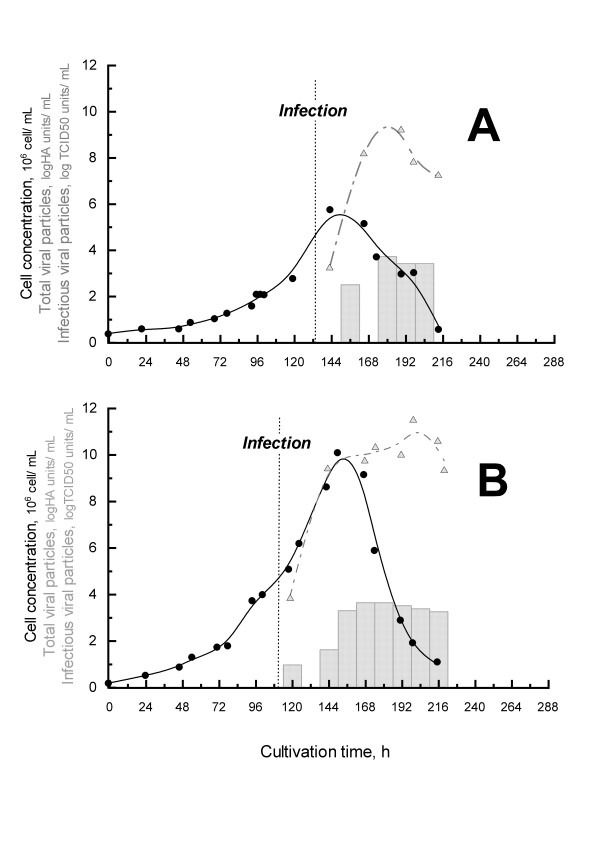
**Influenza production for batch (A) and perfusion (B) cultures**. Viable cells (*black circles*) were plotted with HA (*grey bars*) and TCID50 titers (*up grey triangles*) over time. The perfusion was started at 48 h of culture.

Quantification of the total (HA titers) and infectious (TCID50 titers) influenza particles are presented in Figure 3, table 3 and 4 for both perfusion and batch mode.

**Table 3:** Influenza HA production in batch and perfusion culture at different days post-infection (dpi).

	HA titers, log*HA units/mL *				Total productivity, *logHA units *				Specific productivity, *HA units/10^5 ^cell*			
			
	*1 dpi*	*2 dpi*	*3 dpi*	*4 dpi*	*1 dpi*	*2 dpi*	*3 dpi*	*4 dpi*	*1 dpi*	*2 dpi*	*3 dpi*	*4 dpi*
**BATCH**	2.5	3.7	3.4		6.0	7.2	6.9		6	97	49	

**PERFUSION**	1.9	4.3	4.0	3.8	5.4	7.8	7.8	7.7	0.9	199	191	173

Total viral particles were quantified by hemaglutination assays (HA titers). Total productivity was calculated at different times of harvest post-infection (*Harvest of the perfused flow, 1.5 L/day, was taken into account in the calculation for perfusion system*). Specific productivities were calculated based on total productivity and the maximum cell density attained *(5.76 × 10^6 ^cell/mL in batch and 10.1 × 10^6 ^cell/mL in the perfusion system)*.

**Table 4:** Influenza infectious particle production in batch and perfusion cultures at different days post-infection (dpi).

	TCID50 titers, *TCID50 units/mL*				Total productivity, *TCID50 units*				Specific productivity, *TCID50 units /10^3 ^cell*			
	
	*1 dpi*	*2 dpi*	*3 dpi*	*4 dpi*	*1 dpi*	*2 dpi*	*3 dpi*	*4 dpi*	*1 dpi*	*2 dpi*	*3 dpi*	*4 dpi*
**BATCH**	3.0 × 10^9^	5.3 × 10^10^	3.0 × 10^8^		1.0 × 10^13^	1.6 × 10^14^	7.9 × 10^11^		0.7	10.4	0.1	

**PERFUSION**	2.0 × 10^9^	2.1 × 10^10^	3.3 × 10^11^	2.0 × 10^9^	7.9 × 10^12^	1.6 × 10^14^	1.0 × 10^15^	2.5 × 10^14^	0.3	4.9	36.8	8.5

Total productivity was calculated at different times of harvest post-infection (*Harvest of the perfused flow, 1.5 L/day, was taken into account in the calculation for total productivity in the perfusion system*). Specific productivities were calculated based on total productivity and maximum cell density attained (5.76 × 10^6 ^cells/mL in batch and 10.1 × 10^6 ^cells/mL in the perfusion system).

##### TCID50 detection technique

For the TCID50 titrations, two detection techniques were evaluated and compared in terms of sensitivity and precision. Infected MDCK cell plates were evaluated by microscopy either, with [8] or without immunostaining with antibodies [42]. Detection by immunostaining was clearly more sensitive providing results with a 0.8 log IVP/mL higher titer than the classical microscopic detection technique. A comparison of the results from these two techniques clearly raises the question of the difficulty of comparing influenza viral titers reported in different studies and shows that quantification techniques should be identical to allow sound comparisons. One should carefully and critically evaluate the methods used for titer quantification before drawing conclusions about yields from the published studies. In this study, immunostaining results will be used for comparison with titers obtained in other studies as this detection method is most commonly employed for TCID50 assays [7,8].

##### Viral production

In batch mode, the production kinetics of influenza viruses were very similar to the one observed in previous cultures performed with HEK293 cells [12] or with other adherent cell lines [7,8]. Both HA and TCID50 titers reached a maximal value after 24 hpi, before the start of cell death (Figure 1-C). A maximal total particle titer of 3.7 logHA units/mL and a maximal infectious particle titer of 5.3 × 10^10 ^IVP/mL were obtained at 2 days post-infection (dpi). The total productivity obtained in our 3 L working volume bioreactor was 1.6 × 10^14 ^infectious influenza particles corresponding to 7.2 logHA units (Table 3 &4). In comparison, these results are clearly among the highest values reported in the literature for cell lines such as MDCK, Vero and PER.C6 cells.

Using perfusion mode at a rate of 0.5 vol/day after 48 h of batch culture, allowed a 10-fold increase of the production titers in terms of infectious viral particles. Indeed, the maximal titer obtained was 3.3 × 10^11 ^IVP/mL at 3 dpi in perfusion mode versus 5.3 × 10^10 ^IVP/mL at 2 dpi in the batch mode (Table 4). The increase was less obvious for HA titers, with a 0.6 logHA units/mL increase in perfusion mode (Table 3). The maximal total productivity in the perfusion cultivation, which takes into account the harvest of spent medium (corresponding to 9 L volume in total), was of 1.0 × 10^15 ^IVP and of 7.8 logHA units. This corresponds to a production increase of 8.4 × 10^14 ^IVP and 4.8 × 10^7 ^HA units compared to the batch culture. The significant increase in virus yield could first be attributed to the increase in cell density after infection in the perfusion system (Figure 1C-D). Cell growth was maintained for approximately 32 hpi in this system, reaching a cell density of 10 × 10^6 ^cell/mL. So, in comparison to the batch culture at 48 hpi, 4 × 10^6 ^additional viable cell producers per mLcontributed to the accumulation of viral particles in the culture.

Perfusion also allowed us to maintain or increase the specific productivity of the cells as compared to the batch mode at 2 dpi (Table 3 &4). Thus, at 3 dpi, HEK293SF cells under perfusion mode had a 4-fold higher specific productivity in terms of influenza infectious particles than cells infected in the batch culture mode. This could be attributed to the maintenance of a higher viability and better physiological state of the cells, as explained earlier, by providing fresh medium and removal of potential inhibitors during perfusion.

Furthermore, the daily harvesting of virions in the perfusion system was also a means of preventing further degradation of the particles and thus maintaining a higher productivity. It should be underlined that the total particle titers (logHA units) were more stable than the infectious particle titers (IVP) after 2 dpi, as influenza infection viral particles show a 2-log decrease after attaining their maximal values either in batch or perfusion culture. Degradation of infectious influenza particles in HEK293SF cell culture supernatant has already been observed in the study of Le Ru *et al*. (2010). [12]. This decrease might be explained by the exposure of the virus to the culture temperature or to proteases released in the culture supernatant upon cell lysis over time. The susceptibility of influenza viruses to temperature has already been demonstrated as strain-specific [43] and the A/PR/8/34 influenza strain was shown to be unstable at 50°C [44]. But to our knowledge, no systematic studies addressed the effects of temperature on virus stability within broad operating ranges, and no data is available for the range of temperature usually used for the production.

Overall, as previously reported by Genzel *et al*., (2009), it remains very difficult to compare influenza production performances considering the wide range of cell lines, influenza viral strains, strain variants and production methods as well as the complexity of the cell/virus interaction process [5]. For example, our results from a comparative study showed that the production yields for an A/PR/8/34 strain after three passages in HEK293 cells remains 2-log lower in HEK293 as compared to MDCK cells with infectious titers corresponding to 6.4 × 10^8 ^and 2.1 × 10^10 ^TCID50 units/mL respectively [12]. Furthermore, Schulze-Horsel *et al*. (2009) reported a variation of 1 logHA units/mL for MDCK cell production of A/PR/8 variants obtained either from NIBSC or from the Robert-Koch Institute. They concluded that A/PR/8/34 variants could differ in replication kinetics, yields and eventually in ratio of non-infectious to infectious particle [34]. It is also well accepted that the various influenza strains could have different production yields in the same cell line [9,34,45]. In a previous study we demonstrated that various influenza strains replicate in HEK293 cells and that the H1N1 A types (A/PR/8/34 and A/WS/33), the H3N2 A (A/Aichi/2/68 and A/HongKong/6/68) and a B type (B/Lee/40), were produced at different yields in terms of infectious particles and HA titers [12].

Nevertheless, compared to the data available in the current literature, the values obtained in this study with the perfusion culture of HEK293SF cells are among the highest titers obtained for cell-culture based influenza productions and for both infectious particles (10^7 ^IVP/mL for MDCK cells, 10^10 ^IVP/mL for PER.C6, 10^9 ^IVP/ml for Vero cells) and HA titers (4.0 logHA units/mL for MDCK cells, 4.3 logHA units/ml for PER.C6, 4.0 logHA units/mL for Vero cells) [5]. These values confirm that a HEK293SF cell platform is an excellent alternative for the production of influenza virus compared other cell line platforms, but more importantly, underline the potential in further improving the production yield to optimize the cost-effectiveness and meet surge capacity criteria.

##### Stability study of influenza viral particles in HEK293SF cultures

**Figure 4:**
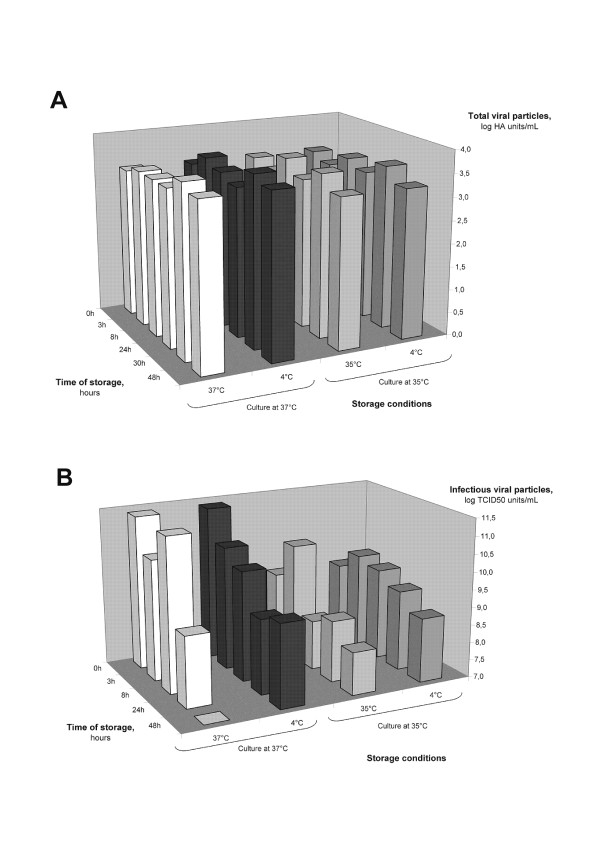
**Stability of influenza total particles (A) and infectious particles (B) at different storage temperatures**. Supernatants from infected HEK293SF cell culture were sampled at 2 dpi and stored at 4°C or at their respective culture temperature (35°C or 37°C) for 48 h.

A stability study was performed to confirm the assumption that perfusion with continual harvest and storage of the production at 4°C allows for a better conservation of the viral particles. Culture supernatants were collected at 2 dpi, for two viral productions performed either at 35°C or 37°C. Samples were then stored either at 2-8°C or kept at the production temperature set point. Results from these experiments confirmed that the total HA content was stable, indicating that the physical (or total) influenza viral particles number was maintained whatever the temperature of storage applied (Figure 4-A). The variations in HA titer observed were lower than the HA assay standard error (0.18 logHA units/mL). In contrast, infectious particles were more affected by storage at high temperature (35°C and 37°C), as a 2-log and a 4-log decrease were observed after 48 h of storage at 35°C and 37°C, respectively (Figure 4-B). Storage at 2-8°C for productions performed at 37°C did not significantly reduce viral particle degradation as a 3-log decrease was still observed. In contrast, for the productions performed at 35°C, storage at 2-8°C reduced the degradation of infectious particles by 1-log. Overall, these results confirm that operating the production at a temperature of 35°C and continuously harvesting the supernatants for subsequent storage at 2-8°C significantly contribute increasing the viral titers and the final yield and justify the perfusion production strategy used in this study.

## Conclusions

The perfusion system is a powerful means to observe the impact of HEK293SF cell metabolism on the influenza infection process and production kinetics. The objectives were to identify if the cell density effect observed beyond 4 × 10^6 ^cells/mL could be overcome by providing a constant supply of nutrients to the cell and eliminating toxic elements from the culture supernatant. This study demonstrates that a significant increase of total and infectious viral particles can be achieved with a perfusion rate of 0.5 vol/day, leading to a 4-fold increase of the viral specific cell production. This productivity gain appears related to the sustained cell growth and the active metabolic state of the HEK293SF cells after infection in the perfusion culture. Overall, this work confirmed that the HEK293SF cell platform is an excellent alternative for the production of influenza virus; compared to other proposed cell lines this cell line has been very well-documented, especially for other viral vector productions and biopharmaceuticals for clinical applications. Because the titers obtained with this cell line are among the highest reported in the literature, this production method could increase the cost-effectiveness and the production capacity of cell culture-based manufacturing of influenza vaccines over other technologies.

## Methods

### Cell lines and culture conditions

The HEK293SF-3F6 cell line that was used was adapted to suspension and serum-free culture [46]. HEK293 cells were cultivated at 37°C and 5% CO_2 _in the serum- and animal-component free medium HyQSFM4Transfx293™ (HyClone, Waltham, MA, USA).

For TCID50 assays, adherent MDCK cells, obtained from the American Type Culture Collection (ATCC CCL-34, Manassas, VA), were cultivated in T75-Flasks containing Eagle's Minimum Essential Medium (EMEM, ATCC) with 10% fetal bovine serum (FBS) at 37°C and 5% CO_2_.

### Cell counts and cell population repartition

For both cell lines, viability and cell density evaluation were performed using erythrosine B dye exclusion on a hemacytometer, with a standard deviation of 10% on the cell counts. Viable, apoptotic and necrotic cell populations were evaluated with the Guava Nexin kit (Guava Technologies Inc., CA, USA) on a LSR II flow cytometer (BD Biosciences, NJ, USA). This kit is using an annexin-V probe to label the phosphatidyl-serines translocated on the external face of the cell membrane during the apoptosis process. The necrotic cells are identified with a 7-AAD probe only able to penetrate porous cells. The accuracy of the cell population quantifications was determined on 3 additional flask cultures (40 mL) infected with A/PR/8/34 at a cell density of 2 × 10^6 ^cells/mL. The maximal standard deviations for each population during the whole culture (respectively 8.8% for viable cells, 6.6% for necrotic cells and 7.0% for apoptotic cells) were used for the bioreactor culture data.

### Bioreactor cultures

#### Bioreactor set-up

3.5-L Chemap type SG bioreactors (Mannedorf. Switzerland) were used for both batch and perfusion cultures. The bioreactor set-up for batch and perfusion operations was previously published (batch [12], perfusion [17,47]). Bioreactors were seeded at 0.25 × 10^6 ^cells/mL in SFM4 Trans Fx293™ (HYQ) medium, and samples were taken twice a day for subsequent analyses. The agitation rate was set to 80-85 rpm, and the dissolved oxygen concentration, pH and temperature were controlled at 40% of air saturation, 7.1 and 37°C, respectively. An additional system to monitor biomass through permittivity (Biomass 400) was installed on the bioreactors (Fogale Nanotech, Nimes, France). Aeration was performed either by surface aeration using a gas mixture of nitrogen and oxygen (gas flow rate of 300 standard cubic centimeters per minute (sccm)) or by sparging pure oxygen in pulse mode when cell concentration was higher than 4 × 10^6 ^cells/mL. In perfusion cultures, cells were grown in batch mode for two days prior to the start of perfusion at 0.5 vol/day. The cells were retained in the reactor with a 10-L acoustic filter operated in a backflush mode. The working volume was 3-L for batch and was set at 2.7-L during the perfusion mode of culture. Each culture was performed once.

#### Infection of HEK-293SF cells

The A/PR/8/34 influenza virus strain (H1N1) was used for the culture infections. The viral stock (10^9 ^IVP/mL) was already produced in HEK293SF cells [12] and was originally derived from a stock obtained from the Global Bioresource Center ATCC. Cultures were infected with influenza viruses at 6 × 10^6 ^cells/mL at an MOI of 10^-3 ^with addition of TPCK trypsin (1 μg/mL) (Sigma, St. Louis, MO, United States), without medium exchange prior to the infection. During the infection phase, the temperature was controlled at 35°C, whereas all other parameters were controlled at standard values throughout the whole culture, as reported in the previous study.

### Metabolite quantification and metabolic parameter calculation

Metabolites were analyzed in the culture supernatant. Glucose, lactate and ammonia concentrations were quantified using an IBI Biolyzer Rapid Analysis System (Kodak, New Haven, US), with standard deviations of 7%, 12% and 33% respectively. Amino acid concentrations were evaluated by HPLC using the Waters AccQ•Tag™ method and the Waters Alliance system (Waters, Milford, MA, US) [48]. This technique is based on amino acid derivatization with a borate buffer and 6-aminoquinolyl-N-hydroxysuccinimidyl carbamate at 40°C during 70 min prior to analysis with a dedicated reversed-phase column (Waters, Milford, MA, US). The variability of this amino acid analytical method is of 10% except in the case of cysteine for which the standard deviation is of 20%.

Depending on the cultivation mode, the specific rates of cell growth (μ), glucose and glutamine consumption (ν_Glc_, ν_Gln_) and lactate or ammonia production (q_Lac_, q_NH3_) were calculated using the following equations. Metabolic yields were then calculated to evaluate the efficiency of each culture mode in term of cell metabolic state.

Batch:

$$\mu =\left(\frac{X_{t+1}-X_{t-1}}{2\times X_{t}}\right)$$

$$\nu =\frac{\left(S_{t-1}-S_{t+1}\right)}{X_{t}}$$

$$\pi =\frac{\left(P_{t+1}-P_{t-1}\right)}{X_{t}}$$

Perfusion:

$$\mu =\left(\frac{X_{t+1}-X_{t-1}}{2\times X_{t}}\right)$$

$$\nu =\frac{\left(S_{t-1}-S_{t+1}\right)+\left(S_{0}-S_{t}\right)\times Q}{X_{t}}$$

$$\pi =\frac{\left(P_{t+1}-P_{t-1}\right)+P_{t}\times Q}{X_{t}}$$

$$Y_{P∕X}=\frac{\pi }{\mu }$$

$$Y_{S∕X}=\frac{\nu }{\mu }$$

$$Y_{P∕S}=\frac{\pi }{\nu }$$

X: cell density

S: concentration of substrates, mM

P: concentration of products, mM

t: cultivation time

Q: perfusion rate

Considering these equations and applying the following equations, it was possible to provide variability values on the metabolic yields calculated.

$$with\;c=a∕b\;;\;so\;\frac{\Delta c}{c}=\frac{\Delta a}{a}+\frac{\Delta b}{b}$$

### Virus quantification

#### TCID50 assays

Infectious viral particles were quantified by the same method as described by Le Ru et al.(2010) [12]. MDCK cells were cultured in EMEM supplemented with 10% FBS in 96-well plates at a seeding cell density of 2.8 × 10^4 ^cells/well. Cells were infected with serial dilutions of culture supernatant after being washed twice with PBS. Supernatant dilutions were performed in 96-well plates with a 5-fold ratio of EMEM medium containing TPCK trypsin (1 μg/mL) at 35°C. TCID50 titers were then evaluated either from microscopic visual detection of plaques assays after 7 days [42] or from microscopic detection of HA protein with antibody fluorescent labeling after two days post-infection [49]. TCID50 titers were then calculated according to the method of Spearman-Karber [50]. Infectious titers that were calculated from the TCID50 assay were expressed as infectious viral particle per mL (IVP/mL). The standard deviation obtained for this assay was 0.5 logTCID50 units/mL.

#### Hemagglutination assay

HA sample contents were quantified by hemagglutination assay using chicken red blood cells, set at 2.0 × 10^7 ^cell/mL. The assays were performed using the protocol described by Genzel *et al*. (2007) [49]. The standard deviation determined for this assay was of 0.18 log HA units/mL.

## Authors' contributions

EP conceived the experiments, carried out the operations and sampling of batch bioreactor cultures, the stability studies, realized the overall data treatments and has drafted the manuscript. VL performed the operations and sampling of the perfusion cultures. DJ and SL supported EP and VL for the preparation of the bioreactors and the cultures control. SA took also a part in the perfusion bioreactor preparation, was involved in the experimental planning and in the editing of this document. AK has been involved in the experiments planning, the drafting of the manuscript and for revising it critically for content and general supervision of the project. All the authors have read and approved the final manuscript.
